# Remove the “Strict” From “Restriction”: A #NephJC Editorial on SODIUM-HF

**DOI:** 10.1016/j.xkme.2023.100615

**Published:** 2023-02-13

**Authors:** Sheikh B. Khalid, Rachel Hung, William Andres Vasquez Espinosa, Nayan Arora, Joel M. Topf, Jamie Willows

**Affiliations:** 1 Department of Internal Medicine, The Indus Hospital, Lahore, Pakistan; 2 King's Renal Unit, King’s College London, London, United Kingdom; 3 Department of Internal Medicine, Saint Peter’s University Hospital, New Brunswick, New Jersey; 4 Division of Nephrology, University of Washington, Seattle, Washington; 5 Department of Medicine, Oakland University, William Beaumont School of Medicine, Rochester, Michigan; 6 Renal Medicine, Sunderland Royal Hospital, Sunderland, United Kingdom

*#NephJC is a recurring twitter-based journal club. #NephJC editorials highlight the discussed article and summarize key points from the NephJC TweetChat.*

## Background

Dietary sodium restriction is commonly recommended to ambulatory patients with heart failure by clinicians worldwide, in the hope of preventing volume overload and improving excess cardiovascular mortality. However, the evidence base for this practice is limited, with a recent meta-analysis demonstrating heterogeneity and highlighting the small sample sizes in individual randomized controlled trials on this topic.^1^ Of concern are some observational studies indicating harm from sodium restriction,^2^ theoretically secondary to increased neurohormonal activation.^3^

Guidelines from Heart Failure Society of America have abandoned the weak recommendation for <2,000 mg/d sodium intake in moderate heart failure^4^ in favor of a 2a-level recommendation to “avoid excessive sodium intake.”^5^ However, the level of evidence is grade C.

The SODIUM-HF trial was designed to advance the low quality and often conflicting evidence around sodium restriction for ambulatory patients with heart failure.

## The Trial

SODIUM-HF was a pragmatic, international, open label, randomized controlled trial conducted at 26 centers across 6 countries. Adults with chronic heart failure (New York Heart Association [NYHA] functional class II-III) and receiving optimal medical therapy were recruited, beginning in 2014. Those who had an average dietary sodium intake of <1,500 mg/d, a serum sodium concentration of <130 mmol/L, or an estimated glomerular filtration rate <20 mL/min/1.73 m^2^ were excluded.

**Figure 1:**
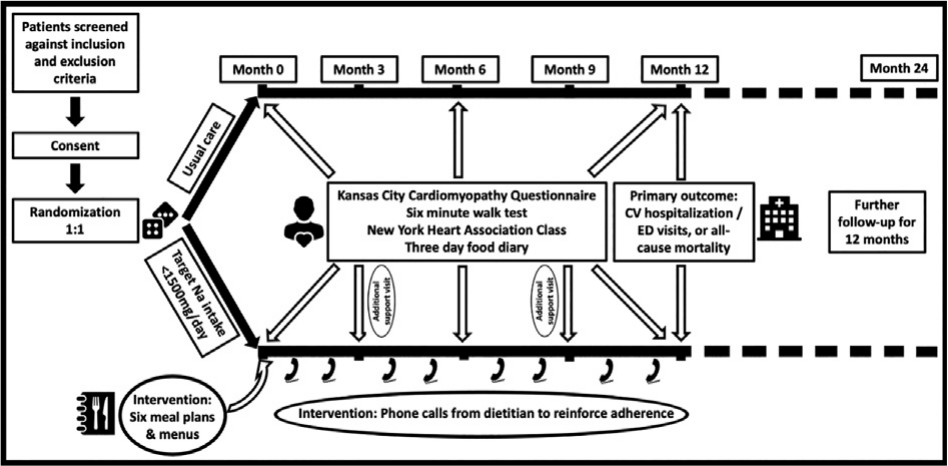
Study schema of SODIUM-HF. Abbreviations, CV, cardiovascular; ED, emergency department.

The trial design is outlined in Fig 1. Participants were randomized 1:1 to either usual care or intensive counseling regarding a low-sodium diet to a target sodium intake of <65 mmol/d (<1,500 mg/d). The intervention included provision of locally adapted menu plans, behavioral counseling, and telephone calls from dietitians to reinforce education and dietary adherence. The control group was given general advice about dietary sodium restriction during routine clinic visits. Dietary sodium intake was assessed with a 3-day food diary.

The primary outcome was a composite of all-cause mortality plus cardiovascular-related hospitalization or emergency department visits within 12 months of randomization. Secondary outcomes included change in the quality of life score, change in 6-minute walk distance, and change in NYHA functional class. Based on an estimated event rate of 25% in the control arm, it was estimated that a sample size of 992 participants would give 80% power to detect a 30% reduction in the primary outcome. However, the trial was stopped early on the advice of the data review committee after a planned interim analysis when 500 patients had completed the 12 month follow-up, on the basis of futility and operational feasibility issues relating to the coronavirus disease 2019 pandemic.

Eight hundred and six patients were enrolled (n = 397 in the low-sodium group and n = 409 in the usual care group). The median age was 67 years, and the mean ejection fraction was 35%. The median baseline sodium intake was similar between both groups; 2,286 mg/d in the low-sodium diet group, and 2,119 mg/d in the usual care group. Over the course of the trial, there was a 28% decrease in reported sodium intake in the low-sodium group to 1,658 mg/d, compared with only a 4% decrease in the usual care group at 2,073 mg/d.

At 12 months, 15% of participants in the low-sodium diet group developed the primary outcome, compared with 17% of participants in the usual care group (hazard ratio, 0.89; *P* = 0.53). Within secondary outcomes, there was a statistically significant but clinically insignificant improvement in quality of life scores in the low-sodium arm, and these participants were more likely to improve one NYHA class than the usual care group (odds ratio, 0.59; *P* = 0.006). There was no difference in the 6-minute walk distance at 12 months and no difference in blood pressure, body weight, caloric intake, or potassium intake between groups.

## The TweetChat

There were 92 active participants who tweeted 597 times when SODIUM-HF was discussed on July 26 and 27, 2022, in North American and Asian time zones, respectively. Given participants were largely nephrologists, the discussion started with the kidney’s role in heart failure, with activation of the renin-angiotensin-aldosterone system and a reduction in water and sodium chloride excretion being important contributors.

It was agreed that the trial question was important to patients and their physicians and that the authors should be congratulated on producing by far the largest randomized controlled trial on the topic. Chat participants have seen how challenging it can be for patients to follow a sodium restriction and worry about decreased quality of life from being unable to eat socially and the development of other nutritional deficiencies from the diet. The pragmatic design and low-cost and scalable intervention helped the study’s applicability to real-world practice. However, SODIUM-HF did not include in their consort diagram how many patients were screened for the study, potentially limiting the generalizability of the results.

An important ambiguity was noted in the paper, as it appears that the authors made a mistake when converting sodium from mg to mmol. The trial title itself states that the reduction of sodium intake was to target <100 mmol/d (equal to 2,300 mg sodium/d), but the paper then discusses aiming for a sodium intake <1,500 mg/d (equal to ∼65 mmol/d). Given that baseline intake was already below the former, it is assumed that the intervention group was targeting the latter, but it was agreed that the *Lancet* editors should provide clarity.

Almost three-quarters of the study population were NYHA class II, with a mean estimated glomerular filtration rate of approximately 60 mL/min/1.73 m^2^ and N-terminal pro–brain natriuretic peptide approximately 850 pg/mL. No patients had an estimated glomerular filtration rate <20 mL/min/1.73 m^2^ or NYHA functional class IV. Chat participants felt that this made the patients relatively low risk for bad outcomes, and a low-risk population enrolled in an underpowered trial makes seeing benefits from any intervention exceedingly difficult. When SODIUM-HF began recruitment in 2014, the definition of “optimal therapy” did not include sodium/glucose cotransporter 2 inhibitors, which have since gained strong evidence of benefit in heart failure,^6^ so it was felt that now these patients would be at lower risk still.

The TweetChat participants acknowledged that the effort had been put into the intervention to try to achieve separation in sodium intake. However, there was disappointment that testing the intervention’s success was reliant on a 3-day food diary rather than urinary sodium testing, which was thought to be less reliable. The sodium restriction arm knew that they were meant to be achieving less sodium intake, so there was concern about social desirability bias creating a false impression of separation of intake between arms. Despite this, the absolute difference in the sodium intake between the groups was reasonably limited at 18 mmol/d, and it is unclear if a larger gap had shown disparate results.

The control group had a reported sodium intake of close to 2,000 mg at 12 months, which is already at the level recommended by some physicians, but this low baseline sodium intake was not thought to represent many of the patients whom chat participants see in clinic. It was hypothesized that if patients with truly high sodium intake had been recruited instead that this may have increased the likelihood of a positive primary outcome and justified guidelines recommending sodium restriction.

SODIUM-HF also assessed clinical outcomes based on prespecified subgroups, where it was seen that participants aged less than 65 years may have derived some benefit from the intervention, but this was considered by most to be “hypothesis generating” at best. Surprisingly another confusing number was noted, as the authors examined the results of a group of patients with baseline sodium intake of <1,500 mg/d, although this was one of the exclusion criteria.

Despite concerns about study power and separation, the study showed an improvement in NYHA class in the intervention arm but without a change in blood pressure, weight, or 6-minute walk test between arms. Chat participants wondered whether the more subjective measure of NYHA class was confounded by performance bias from additional follow-up visits and the unblinded nature of the trial. Diuretic use was not reported, but if superior volume control was indeed present in the intervention arm, it was felt this could be equally achieved by diuretic prescription and that this strategy may be preferred by patients over long-term dietary alterations.

At the conclusion of the journal club, most nephrologists felt that they would continue to counsel against excess sodium for their patients with heart failure but that the results of SODIUM-HF had not persuaded them to change their current practice.

## Conclusion

For patients with heart failure and baseline sodium intake of 2,000 mg/d, the largest randomized controlled trial of further sodium restriction using dietary counseling methods did not show improvement in hard clinical end points. A keen focus on optimizing guideline-directed medical therapy will continue to be foremost in improving outcomes for these patients.
